# ﻿A new dioecious bush tomato, *Solanum nectarifolium* (Solanaceae), from the northern Tanami Desert, Northern Territory, Australia, with reassessment of *S. ossicruentum* and a change in the circumscription of *S. dioicum*

**DOI:** 10.3897/phytokeys.268.169893

**Published:** 2025-12-29

**Authors:** Christopher T. Martine, Kym Brennan, Jason T. Cantley, Aiden T. Webb, Geoffrey Newton

**Affiliations:** 1 Department of Biology, Bucknell University, 1 Dent Drive, Lewisburg, PA, USA Bucknell University Lewisburg United States of America; 2 Northern Territory Herbarium, Department of Lands, Planning and Environment, Palmerston, Northern Territory, 0830, Australia Northern Territory Herbarium Palmerston Australia; 3 Department of Biology, San Francisco State University, 1600 Holloway Avenue, San Francisco, CA 96132, USA San Francisco State University San Francisco United States of America

**Keywords:** Ant–plant mutualism, Australia, coadaptation, dioecy, extrafloral nectaries, inaperturate pollen, new species, Northern Territory, plant–animal, Tanami Desert, Winnecke Hills

## Abstract

A new species of functionally dioecious bush tomato from the “*Solanum dioicum* + *S. echinatum* group” of Solanum subgenus Leptostemonum is described. *Solanum nectarifolium* Martine & Brennan, **sp. nov.**, is a member of the taxonomically challenging *Solanum dioicum* W.Fitzg. species complex in Australia and differs from other species in the group by the presence of prominent and conspicuous extrafloral nectaries on the abaxial leaf surfaces, a tinge of purple on the new growth and young fruiting calyces, and curved prickles on young stems. Although fairly well represented in herbarium collections, this new taxon has historically been treated as a part of *S. dioicum* and more recently as a part of *S. ossicruentum* Martine & J.Cantley due to shared vegetative characters, particularly a silvery-blue aspect. Symon’s “Group three” or “Tanami form” of *S. dioicum* is thus now treated as two species: *S. nectarifolium* (with prominent nectaries, a deeply bifid stigma, and an exposed berry at maturity) and *S. ossicruentum* (lacking visible nectaries and possessing a slightly lobed and nearly linear stigma, plus a berry fully enclosed in the calyx at maturity). While extrafloral nectar production has been documented in *Solanum*, *S. nectarifolium* is the first known example in the genus with nectaries that are consistently visible to the naked eye. This new species brings the number of functionally dioecious (and morphologically androdioecious) *Solanum* species in Australia to 15. We also provide an updated distribution for *S. ossicruentum* and, in light of our findings, reassess the identity of specimens associated with the type series for *S. dioicum*.

## ﻿Introduction

The Australian Monsoon Tropics (AMT) is a center of recent and ongoing plant diversification shaped by climatic fluctuations and a mosaic of sandstone landscapes that promote ecological heterogeneity (Bowman et al. 2010; Edwards et al. 2017, 2018). Particularly high levels of plant diversity occur within the AMT, and recent decades have witnessed near-continuous publication of newly described species and phrase-named taxa from the region. This trend toward diversification is readily apparent in the variety of forms seen in the “*Solanum dioicum* + *S. echinatum*” clade (as per Martine et al. 2019) of the Leptostemonum clade (the so-called spiny solanums), as some taxa appear to have become narrowly endemic in refugial sandstones (e.g., *S. carduiforme* F.Muell.), while others seem to have expanded into sandstone-associated savannah and open woodland habitats (e.g., *S. dioicum* W.Fitzg.). Recurring periods of habitat retraction and expansion are hypothesized to have manifested in the present day as the complex variety of forms seen among *Solanum* taxa occurring across the AMT. *Solanum dioicum*, as defined by Symon (1981), is an especially problematic species complex, with perhaps 20+ recognizable variants (M. and R. Barrett, pers. comm.) occurring primarily in the Kimberley region of Western Australia and the Top End region of the Northern Territory.

In this paper, we aim to resolve two sources of confusion. First, we describe *Solanum nectarifolium* Martine & Brennan, sp. nov., a morphologically and phylogenetically distinct taxon previously included in *S. dioicum* sensu lato and later considered part of *S. ossicruentum* Martine & J. Cantley. Second, we revisit the typification of *S. dioicum* itself, given the previous inclusion of specimens now attributable to *S. ossicruentum*.

*Solanum dioicum* is part of the “*S. dioicum* + *S. echinatum*” clade, with ca. 45 currently recognized species and numerous morphological variants exhibiting different sexual systems. Species in this clade may be cosexual (producing only cosexual flowers, as in *S. echinatum* R.Br.), andromonoecious (bearing inflorescences typically consisting of a single cosexual flower and a set of distal staminate flowers, as in *S. chippendalei* Symon), or functionally dioecious. The latter sexual system, found in *S. dioicum* and related taxa (see Anderson and Symon 1989; Brennan et al. 2006; Martine 2023), can be a challenge to recognize in the field: unisexually “male” plants bear easily interpreted cymes of staminate flowers, but functionally “female” plants bear solitary flowers that appear to be cosexual but are rendered functionally female via the production of inaperturate pollen that cannot germinate. Because only one sex is often represented in a collection, and because floral morphology does not always reflect sexual function, sexual systems are sometimes misinterpreted. For non-specialists in particular, this can lead to substantial confusion, with specimens misidentified, misfiled, or inconsistently annotated in herbaria—ultimately masking taxonomic diversity and complicating efforts to describe it.

Earlier phylogenetic work (Martine et al. 2006, 2009, 2019) divided the dioecious Australian solanums into two major lineages: the “Kakadu” and “Kimberley” clades—the latter of which includes a set of *S. dioicum* forms associated primarily with sandstones in the Kimberley region and western coast of northern Western Australia (WA), with extensions east into the Top End of the Northern Territory (NT). Since its collection by W. Fitzgerald in 1906 (Fitzgerald 1918), *Solanum dioicum* has historically served as a catch-all for a diverse array of forms spread across much of northern Australia. Work to better define the differences among the variants of *S. dioicum* and the boundaries among them is ongoing (e.g., Marino 2023; M. Barrett, in prep.; McDonnell and Martine, in prep.; Marino et al., in prep.). In the meantime, it is still useful to recognize and name entities that are clearly distinct (such as *S. scalarium* T.Williams et al.; Williams et al. 2022) as both a means to reduce confusion and to ensure that these taxa can be properly assessed as conservation management units.

Symon, in his 1981 monograph on Australian *Solanum*, defined three morphological groupings of *S. dioicum* based on his fieldwork and the relatively limited number of specimens available at that time. One of these groupings, his “Group three—inland and Tanami,” was distinguished as a set of populations occurring “mainly inland and on the eastern margin of the distribution range.” These plants, which have been annotated since that time in collections as “Tanami form” (because of populations that reach the northern edge of the Tanami Desert) or by Symon as the “extreme NT form” (e.g., his 1975 annotation on a specimen of *G. W. Carr 3374* held at DNA), are “closely and densely silvery-pubescent, compact, and extremely prickly.” Among the herbarium specimens cited as being representative of “Group three” were collections from two disjunct geographic regions: the northwestern Tanami region (such as *Symon 6937*, from 60 km SW of Hookers Creek, NT) and the areas north of Lake Argyle (WA) and other specimens from just east of the nearby WA/NT border (such as *Symon 5242*, from 14 km W of East Baines River). All of these collections show the characteristic short indumentum, silvery-blue foliage, and general prickliness described by Symon. The publication of three groups as a single *Solanum dioicum* by Symon (1981) was a historically important step in the iterative process of describing the variant forms found in nature, but it also created some confusion about how W. Fitzgerald intended to define *S. dioicum* when he described the species (Fitzgerald 1918), including the assigning of type specimens.

Based in part on Symon’s specimen citations, populations from across the “Tanami form” range were described as *Solanum ossicruentum* Martine & J.Cantley, formalizing the form as a distinct species (Martine et al. 2016). Martine et al. (2016) combined field observations from three sites north of the Tanami Desert, including Mirima National Park (WA), the Carr Boyd Ranges (WA), and Keep River National Park (NT), with inferences from plants grown in cultivation from wild-collected seed and herbarium sheets held at the Northern Territory Herbarium, Palmerston (DNA).

However, soon after the publication of *S. ossicruentum*, botanists revisiting the somewhat remote collection sites in the southern part of the species range (i.e., populations on the northern edge of the Tanami Desert) noted not only that those populations did not key out correctly, but also that they exhibited a number of morphological differences that appeared to not match the type form as described in Martine et al. (2016), including a deeply bifurcating stigma in the functionally female flowers and the presence of conspicuous, round, green to black dots (apparent extrafloral nectaries) on the undersides of the leaves. Likewise, unpublished phylogenomic results (McDonnell and Martine, in prep.) infer that the southern inland populations represent a distinct lineage not part of *S. ossicruentum*. Here we correct the previous oversights and define the elements that characterize the true “Tanami form” as a species distinct from *S. ossicruentum* and *S. dioicum* sensu lato. The designation of the southern taxon as *Solanum nectarifolium* Martine & Brennan, sp. nov. brings the total number of functionally dioecious (and morphologically androdioecious) *Solanum* species in Australia to 15.

## ﻿Methods

**Figure 1.**
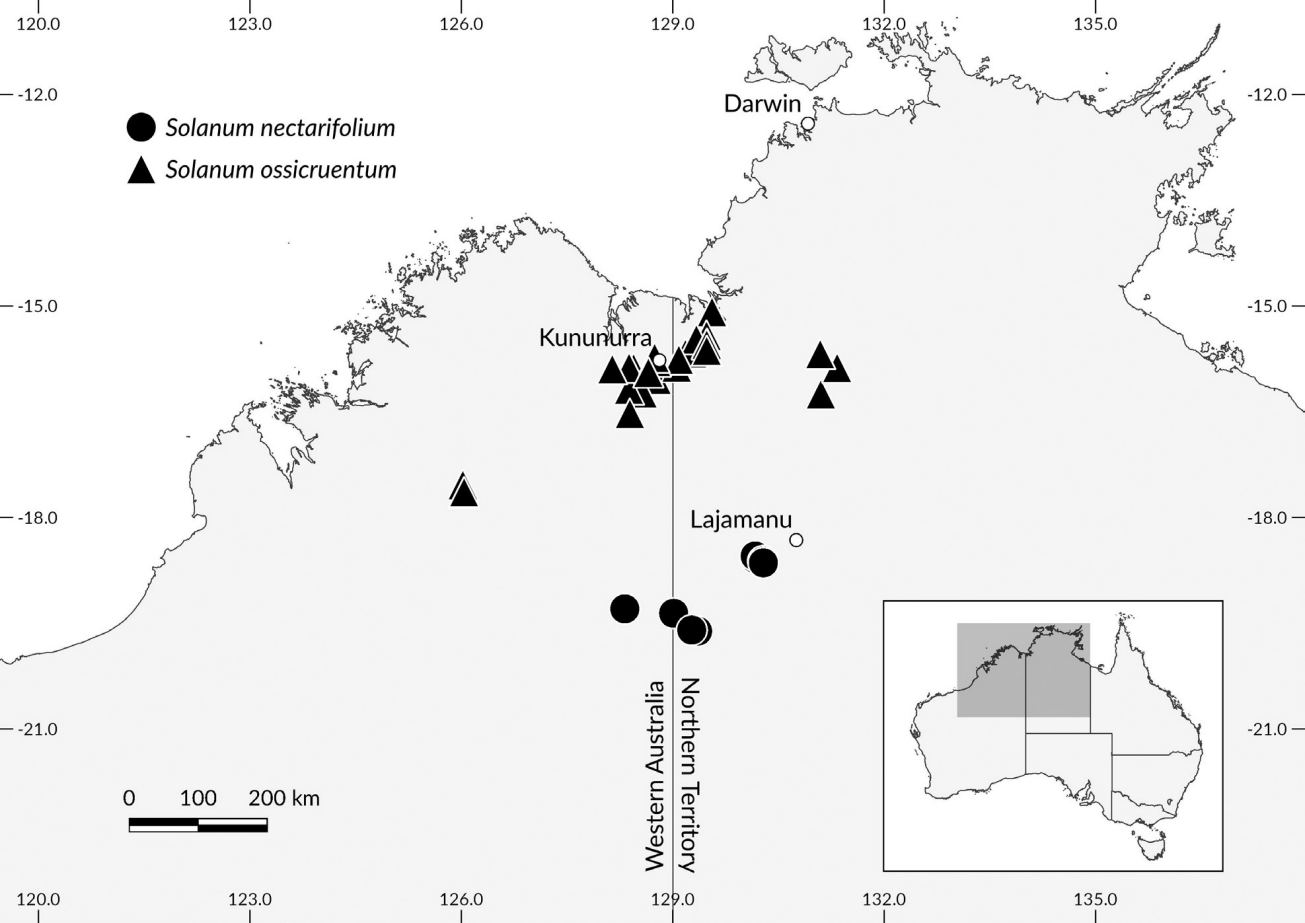
Map showing distribution of *Solanum nectarifolium* sp. nov. and *S. ossicruentum* based on accessions held at the Northern Territory Herbarium, Palmerston (DNA), the Western Australian Herbarium (PERTH), and the National Herbarium of New South Wales (NSW). Map generated in QGIS 3.4.

Recent observations by the authors during May/June 2025 fieldwork in the Winnecke Hills area south of Lajamanu, NT (Fig. 1), are combined here with inferences from herbarium sheets held at the Northern Territory Herbarium at Darwin (**DNA**), the Western Australian Herbarium (**PERTH**), and the National Herbarium of New South Wales (**NSW**). All material filed as *S. dioicum* or *S. ossicruentum* was examined across the three herbaria, with more than 50 sheets confirmed or redetermined to represent either *S. nectarifolium* (Suppl. material 1) or *S. ossicruentum*. A map (Fig. 1) of vouchered occurrences was generated using records from the Australasian Virtual Herbarium and modified based on the current understanding of which specimens are now representative of each taxon. Because the putative type series of original material accessioned at NSW (as holotype and isotypes) for *S. dioicum* included material from two different species rather than clear duplicates of a single gathering, two sheets were redetermined as *S. ossicruentum* and removed from consideration as duplicates.

Measurements included in the species treatment were made in the field using fresh material, supplemented as needed with herbarium material held in the collections cited above.

## ﻿Taxonomic treatment

### ﻿ Solanum nectarifolium

Taxon classificationPlantaeSolanalesSolanaceae

Martine & Brennan sp. nov.

FBF66E47-3147-5228-9A81-452004105DBC

urn:lsid:ipni.org:names:77374377-1

Figs 2, 3

#### Diagnosis.

**Figure 2.**
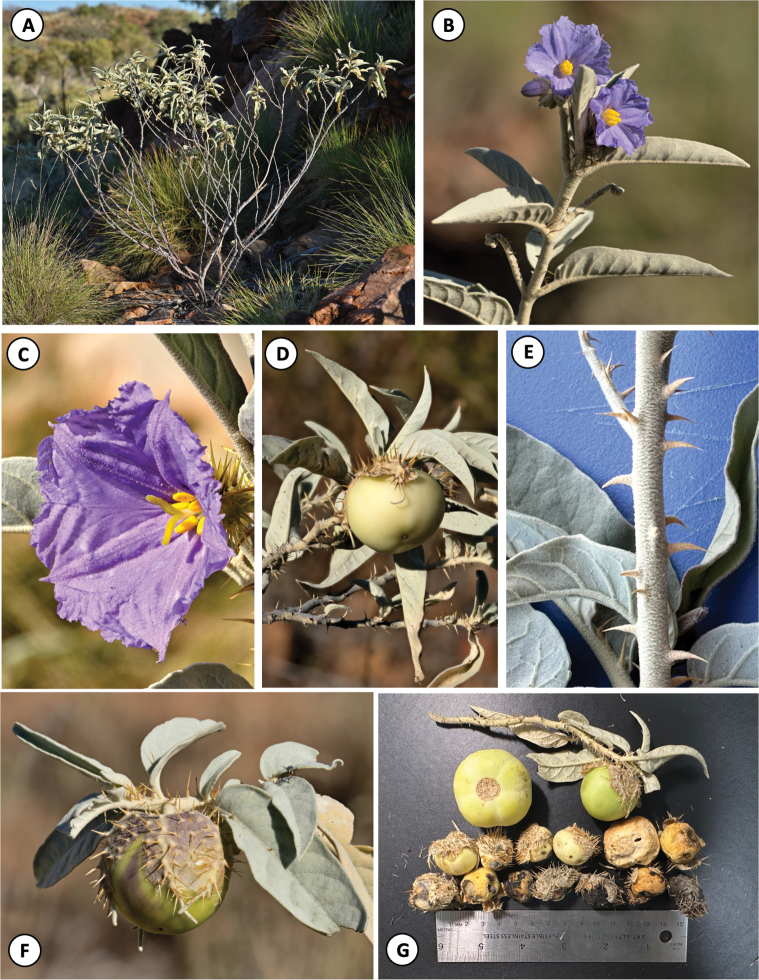
Habit and morphology of *Solanum nectarifolium* Martine & Brennan, sp. nov. **A.** Upright open growth habit; **B.** Staminate flowers; **C.** Functionally female (morphologically cosexual) flower (note bifurcating stigma); **D.** Mature pre-abscission fruit with mature, gray, prickly calyx; **E.** Curved prickles on young stems; **F.** Immature fruit and fruiting calyx (note purple tinge); **G.** Variation in fruit sizes, all fruits in lower rows abscised, collected from ground with persistent calyces, becoming dry, light, and densely polystyrene foam-like in texture. Photos (**A–D, F**) by K. Brennan; (**E, G**) by C. Martine; all photographed material associated with the collection *Martine 5800*.

**Figure 3.**
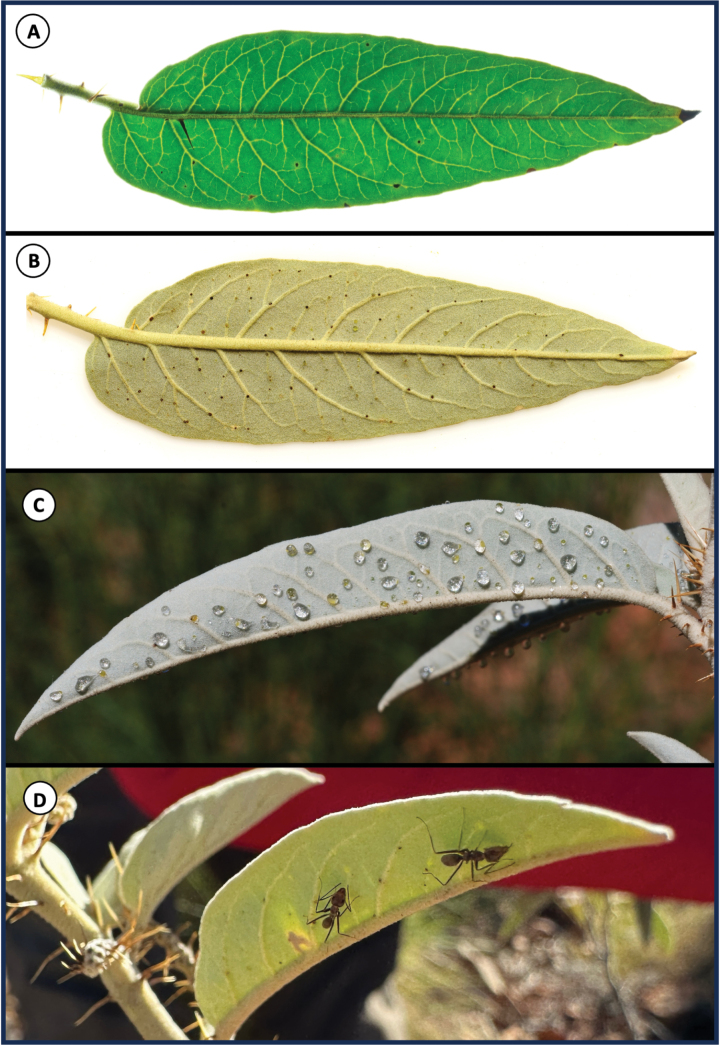
Extrafloral nectaries (EFNs) on the leaves of *Solanum nectarifolium* Martine & Brennan, sp. nov. **A.** Adaxial leaf with backlighting, veins illuminated, prickle on midvein; **B.** Abaxial leaf, natural light, showing raised venation and green to black EFNs on lateral veins; **C.** EFNs exuding extrafloral nectar in nature; **D.** Ants probing EFNs at the type locality. Photos (**A–C**) by K. Brennan (associated with *Brennan12086*), (**D**) by C. Martine (associated with *Martine 5800*).

*Solanum nectarifolium* is distinguished from other taxa in the *Solanum dioicum* W.Fitzg. species complex by the presence of prominent and conspicuous extrafloral nectaries on the veins of the abaxial leaf surfaces (Fig. 3), a tinge of purple on the new growth and young fruiting calyces (Fig. 2F), and curved prickles on young stems (Fig. 2E). It is further distinguished from *Solanum ossicruentum* Martine & J.Cantley by having fruit that is only partially enclosed by the calyx (rather than fully) (Fig. 2D, F, G), a calyx that is far less prickly, and its deeply bifurcating stigmas (rather than only slightly lobed and nearly linear) (Fig. 2C).

#### Type.

Australia • Northern Territory: Winnecke Hills, ca. 50 km SW of Lajamanu on the Lajamanu Road, -18.642361, 130.289194, 5 June 2025 (fl, fr), *C.T. Martine 5800 with K. Brennan, J.T. Cantley, G. Newton, A.T. Webb* (holotype: DNA; isotypes: PERTH, CANB, NY, US, BUPL).

#### Description.

Upright, low-branching, spreading woody shrub to 0.4–1.8 m tall and 1–2.5 m wide. Multistemmed from base or with single woody stem up to 2.5 cm diameter from woody rootstock, producing first branches ca. 5–15 cm above base and ultimately branching ca. 4–10 times. Overall plant aspect silvery to bluish green to gray-green, the young growth tomentose-lanate, with older stems woody and whitish-gray. Stems with short, dense indumentum of stellate trichomes. Prickles on young, yellow-green stems stout, curved, sharp, 3–7 mm long, slightly widened (1.5–2 mm) at base; older stems with prickles scattered to absent, weak, slightly curved, 3–5 mm long, < 1 mm wide at base.

Leaves simple, alternate, 3–12 cm long, 20–32 mm wide, lanceolate, soft silvery-blue. concolorous, both sides densely silvery-tomentose; trichomes mostly short stalked, porrect-stellate with short central ray (midpoint) and 6–8 lateral rays; long-tapering single-celled trichomes and gland-tipped linear trichomes also present and scattered on margins and abaxial surface; abaxial leaf surface dotted with small, rounded, green to blackish extrafloral nectaries ca. 0.5 mm diameter on lateral veins (even those of small order), conspicuously visible without magnification and, when active, exuding a clear, sticky, sweet liquid; abaxial surface with midveins and larger lateral veins raised; margins entire; base truncate to rounded, asymmetrical, consistently oblique by 3–5 mm; petiole 4–7 mm long, with 0–4 prickles 5–6 mm long; upper midveins of adaxial surface with 0–3 prickles 5–6 mm long. Inflorescences borne on new growth, at first terminal, then becoming leaf-opposing; consisting of solitary cosexual (functionally female) flowers and cymes of male flowers on separate plants.

***Staminate*** (functionally male) inflorescence a helicoid cyme ca. 4–5 cm long with 8–16 flowers, unbranched, typically with only 2–3 flowers open at a time and then abscising; buds up to 1 cm long before blooming; peduncle ca. 2–2.5 cm long, sparsely armed; rachis 2–2.5 cm long with 1–5 mm long slightly curved prickles alternating with each flower; pedicels ca. 2 mm long, unarmed, abscising at base; calyx 5-lobed, unarmed, purple-tinged, the lobes 6–7 mm long with linear acumens; corolla 1.7–2 cm (rarely to 3.8 cm) diameter, purple, rotate-stellate to rotate, stamens 5, ca. 5.5 mm long, equal; anthers ca. 5 mm long, oblong-lanceolate to somewhat tapered, bi-cleft, connivent, yellow, poricidal; filaments ca. 4 mm long, connate at base; ovary, style, and stigma vestigial, non-functional, and not exserted beyond the stamens.

Morphologically ***cosexual*** (functionally ***female***) flowers solitary; pedicel 0.25–1.0 cm long, armed with small prickles to 2 mm long; calyx densely armed along ribs of tube with 4–5 mm long, straw-colored straight prickles and stellate trichomes, acumens 10–14 mm, narrowly linear (when in bud extending beyond the tip and crossing over each other like a loosely-tied knot); corolla ca. 4 cm diameter, rotate-stellate to stellate-campanulate/funnelform, blue-violet; stamens of same proportions as in male flowers; anthers assumed to produce inaperturate pollen; style erect, stigma bright green and deeply bifurcated with lobes 5 mm long.

Fruit a berry 2–3 cm (rarely to 4 cm) wide, 2.3–3 cm long, globose, light green when immature, sometimes with a purple tinge; ripening to yellow-creamy with a waxy bloom, then aging from creamy white to brown and eventually black, at first fleshy but firm (almost apple-like); fruit wall at first leathery, fleshy but tough (like dried apple), later fully drying to a firm polystyrene foam-like texture; the whole fruit becoming remarkably light in weight. In early development, fruit nearly fully enclosed in prickly and silvery blue-green (at times purple-tinged) calyx, with long, finger-like acumens clasping around the open end of the ripening fruit, then straightening out post-maturity, calyx tube detaching slightly but remaining enclosed around ca. 40% of the berry; calyx firmly attached at the base of the fruit, aging to papery tan then gray, calyx tube not adhering to the fruit surface but also not detaching from the base. Calyx prickles nearly absent to scattered on the laminar portions, 2–7 mm long; prickles concentrated on the midveins of the calyx tube, 5–7 mm long, 1 mm wide at base, tapering to a long sharp tip. Fruit and calyx together forming a diaspore that (typically) abscises from the plant at maturity, falls to the ground, becomes dried out and light in weight, fruits occasionally remaining on the plant for longer, growing larger and remaining fleshy. Seeds ca. 2 mm long, broadly kidney-shaped, chestnut-brown to nearly black at maturity, sticky and adhering in a single layer on the placental tissue and inner fruit wall.

#### Distribution and ecology.

**Figure 4.**
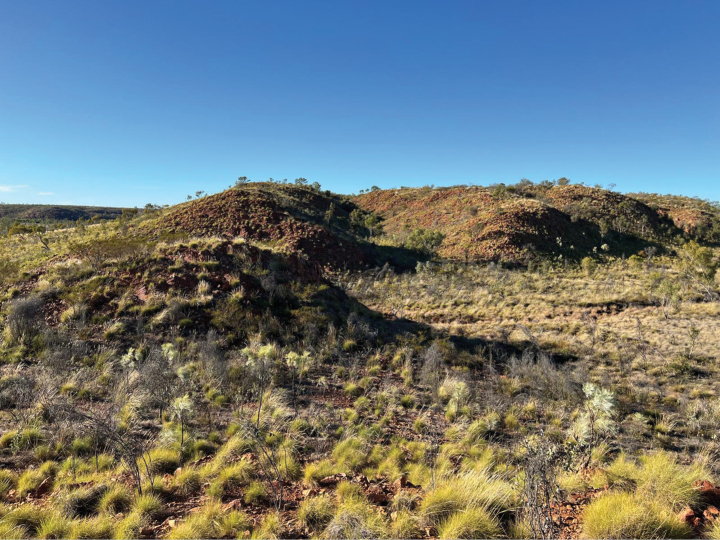
Habitat of *Solanum nectarifolium* sp. nov. at the type locality. Low quartzite sandstone formations at Winnecke Hills, south of Lajamanu, northern edge of the Tanami Desert, Northern Territory. Photo by A. Webb.

*Solanum nectarifolium* is presently known from the northwestern edge of the Tanami Desert, with most collections made in the Winnecke Hills region (Fig. 4) 40–70 km south of Lajamanu, further south along the Lajamanu Road, and west into WA in the Gardner Ranges off the Tanami Road (Fig. 1). The species occurs on hilltops on dissected quartzite sandstone pavement, among boulders and rocks on hillsides, and in sand and gravel of washes and plains between and extending from hills. Many of the collections currently held in herbaria were made along or near the most accessible tracks in the region, but suitable habitat for *S. nectarifolium* extends far beyond where vehicles can easily drive or where botanists have been afforded access. This suggests that *S. nectarifolium* may be more abundant in that localized region than currently understood. However, there is no evidence that it occurs outside of a fairly restricted distribution range, which is wholly disjunct from the more-northern distribution of *S. ossicruentum* (Fig. 1).

The vegetation at the type locality (Winnecke Hills) is a low open woodland dominated by *Blakella aspera* (F.Muell.) Crisp & L.G.Cook (Myrtaceae) and *Eucalyptus brevifolia* F.Muell. (Myrtaceae), with scattered *Hakea lorea* R.Br. (Proteaceae) and a sparse low mid-story including *Grevillea pyramidalis* A.Cunn. ex R.Br. (Proteaceae), *Grevillea wickhamii* Meisn., *Acacia stipulosa* F.Muell. (Fabaceae), *Acacia lycopodiifolia* Hook., *Acacia retivenea* F.Muell., *Acacia acradenia* F.Muell., Hibiscus cf. leptocladus Benth. (Malvaceae), *Jacksonia odontoclada* F.Muell. ex Benth. (Fabaceae), and *Mirbelia viminalis* (A.Cunn. ex Benth) C.A.Gardner (Fabaceae). The sparse ground layer is dominated by hummock grasses, *Triodia spicata* N.T.Burb and *T. bitextura* R.L.Barrett & M.D.Barrett (Poaceae) with Solanum aff. echinatum R.Br. (Solanacaeae), *Tephrosia lasiochlaena* Cowie (Fabaceae), *Triumfetta micracantha* F.Muell. (Malvaceae) and *Cheilanthes brownii* (Desv.) Domin (Pteridaceae).

*Solanum* flowers do not produce nectar; they are buzz-pollinated by bees foraging for pollen. While it is not known what bees visit the flowers of *S. nectarifolium*, bee genera such as *Xylocopa* and *Amegilla* have been recorded on related Australian congeners (see Anderson and Symon 1988; Switzer et al. 2016). It is also likely that *S. nectarifolium* pollen offers differential rewards via the pollen produced by male versus functionally female flowers, as seen in related taxa (Ndem-Galbert et al. 2021).

Our observations of the foliar extrafloral nectaries (EFNs) being fed on and actively defended by ants (Fig. 3) further confirm a plant-insect coadaptation previously suggested for related species by Anderson and Symon (1985) and under study by Henry et al. (in prep). Despite there being other *Solanum* species in the “Kimberley dioecious clade” (e.g., *S. cunninghamii* Benth., *S. tudununggae* Symon, *S. dioicum*) known to produce EFNs (on the leaves, flower buds, and abaxial corolla surfaces), these are only visible under a microscope (Anderson and Symon 1985). By contrast, those of *S. nectarifolium* are large (0.5 mm diam.) and are uniquely, conspicuously visible to the naked eye (Fig. 3B). Foliar and floral EFNs have otherwise rarely been recorded in *Solanum* (perhaps only by Whalen et al. (1981) for Solanum section Lasiocarpa), in part because nectaries are typically microscopic and nectar is quickly consumed by insects as it is exuded (Anderson and Symon 1985; S. Knapp pers. comm.). Lortzing et al. (2016) reported nectar secretion from wounds in *S. dulcamara* L. and Sampaio et al. (2022) described reward-producing resin glands on petioles in *S. fernandesii* V.S.Sampaio & R.Moura, but neither instance represents production of sugar-rich nectar by dedicated organs.

The seed dispersal mechanism for this species is unconfirmed. Young, fleshy, green fruits (Fig. 2F) are mostly enclosed in a prickly calyx that gradually loosens as fruits move from yellow when ripe to later becoming creamy-white, dry, and very light in weight with the texture of firm polystyrene foam (Fig. 2D, G). These lighter fruits with their prickly calyces attached are shed by the plants as apparent trample-burr diaspores, suggesting that endozoochory is less likely than ectozoochory (see Symon 1979; Martine et al. 2019; Motter 2025). Apparently germinable seed, chestnut-brown to black in color in dried-out fallen fruit, was also noted in attached ripened, lemon-yellow, fleshy fruits (*Martine 5800* type collection), suggesting that effective seed dispersal could also happen at this stage. However, seeds within attached green fruits still firmly enclosed in the calyx were white to tan and appeared to be immature.

Localized *S. nectarifolium* recruitment and persistence appears closely tied to fire frequency, given observations made at Winnecke Hills by the group in 2025 and by K. Brennan in 2021. In 2021, plants were abundant west of the Lajamanu Road, where fires had occurred a few years prior, and were compact, densely-branching, and vigorously reproductive (flowers and fruits) plants; east of the road, where fires were very recent, live plants were not conspicuous. In 2025, the eastern populations consisted of hundreds of even-aged mature plants (post-fire recruits from 2021) approaching senescence, and almost no living plants were seen to the west, soon after a new fire that appeared to have killed all mature plants. Because the flammability of habitats in the region is determined mostly by the presence and maturity of *Triodia* grasses, persistence of *S. nectarifolium* appears to be aligned with the burning cycle of *Triodia*. Mature *Triodia* is highly flammable but regenerates slowly once burnt, requiring a few years for hummocks to grow to a point where they will effectively carry another fire. As an apparent obligate seeder (not a re-sprouter), *S. nectarifolium* probably re-establishes from the seedbank in the next season after a fire with adequate rainfall and reaches peak flowering and fruiting during the 1–2 years when *Triodia* fuel is building up. In subsequent years, as the *Triodia* around it becomes increasingly vulnerable to another burn, *S. nectarifolium* (if not burnt) continues to produce diminishing amounts of new seed before eventually senescing.

#### Phenology.

Plants encountered on 6 June 2025, after an average to below-average summer rainfall season, were largely past-bloom, with only one functionally female flower (Fig. 2C) seen across hundreds of plants. Likewise, only a few female plants bore fruits. New flowers began opening at sunrise, were fully open by mid-morning, and started to close and senesce by late afternoon; male flowers appeared to be slightly more ephemeral. Herbarium specimens bearing both flowers and fruits have been collected from February through September. In the week or so prior to our June visit there had been a significant unseasonal rainfall event, and many of the largest hilltop plants, which showed signs of earlier partial senescence, had begun resprouting from the lower portions of the largest lateral branches. This response suggests that the species may be able to respond rapidly to rainfall at any time, a favorable strategy in a semi-arid region where rainfall is often highly unpredictable.

#### Etymology.

The epithet *nectarifolium* is chosen based on the presence of its conspicuous foliar extrafloral nectaries (Fig. 3), a character apparently unique among the 1200+ species of *Solanum*. We suggest the use of Tanami Bush Tomato for the English-language common name of the species in recognition of the Tanami region where all collections have been made and to maintain a connection to the historically-applied “Tanami form” name. We also suggest the English-language common name for *Solanum ossicruentum* as Bloodbone Bush Tomato, the name “bloodbone tomato” having been widely used in media coverage when that species was described.

#### Preliminary conservation status.

Based on IUCN Red List Categories (IUCN 2012), *S. nectarifolium* is considered Data Deficient (DD). Although the species is relatively widespread, its overall status is not well understood, being known from a relatively small number of collections from only a few sites. While the type locality supports a population of hundreds of individuals, its abundance elsewhere has not been recorded. Further data are required to fully assess its current conservation status. A significant existential threat to native plants across central Australia is the potential invasion of buffel grass (*Cenchrus ciliaris* L., Poaceae; Marshall et al. 2012). This introduced perennial pasture grass is widespread and capable of transforming landscapes by completely overwhelming native vegetation through direct competition or by altering fire regimes. It establishes best on substrates with high fertility and elevated pH. There was no buffel grass observed at the Winnecke Hills type locality in 2025 despite herbarium records from sites nearby. However, the types of infertile, pH-neutral quartzite sandstone soils where *S. nectarifolium* occurs have been noted as resistant to buffel grass establishment (Marshall et al. 2012), so any future buffel grass incursion may be less impactful than where its ideal ecological circumstances prevail.

#### *Solanum nectarifolium* specimens (paratypes) examined.

(bolded collectors/numbers are specimens previously cited as paratypes of *S. ossicruentum* by Martine et al. (2016)) **Australia** • **Northern Territory**: 60 km SW of Hookers Creek, 165.8 km NE of Tanami, 18°35'S, 130°10'E, 18 May 1971, *Symon 6937* (staminate material) (PERTH); • 60 km SW of Hookers Creek, 165.8 km NE of Tanami, 18°33'S, 130°10'E, 18 May 1971, *Symon 6938* (fruiting material) (DNA, PERTH); • Waite Institute (pot grown from DES 6938), 17 April 1973, *Symon s.n.* (staminate material) (DNA); • Jellebra Rockhole, 19°21'45"S, 129°00'35"E, 7 June 1996, ***Albrecht 7756*** (fruiting and staminate material) (DNA); • 11 km east of NE Mt. Frederick, 19°37'S, 129°21'E, 1 March 1981, ***Latz 8597*** (staminate material) (DNA); • Pargee Range, 19°36'S, 129°16'E, 2 April 1981, ***Latz 8608*** (fruiting material) (DNA); • Winnecke Hills, 18°37'11"S, 130°16'30"E, 1 May 2004, ***Mangion & Lewis 1607*** (staminate material) (DNA); • 63 km S of Lajamanu, 18.39°S, 130.16°E, 10 Feb 1988, ***Orr 57*** (fruiting/flowering material) (DNA); • 37 km SW Hookers Creek, 18 May 1971, *Maconochie 1122* (staminate material) (PERTH, NT); • 37 km SW Hookers Creek, 18 May 1971, *Maconochie 1121* (fruiting material) (PERTH, NT); • 48 km SW Lajamanu (direct), 18°37'06"S, 130°16'50"E, 15 April 2021, *Brennan 12086* (fruiting/flowering material) (DNA); • Winnecke Hills, 50 km SSW of Lajamanu, 18°38'40"S, 130°07'30"E, 29 September 2003, *Latz 19451* (fruiting material) (DNA, NT). **Western Australia** • Sturt Creek Station, Denison Range, 19°18'S, 128°19'E, 20 July 1973, ***Latz 4019*** (fruiting material) (DNA); • Gardner Range, 50–60 mi. NW of Tanami, August 1971, *Gittins 2396* (staminate/fruiting material) (NSW); • Gardner Range, 50–60 mi. NW of Tanami, August 1971, *Gittins 2401* (staminate/fruiting material) (NSW).

#### *Solanum ossicruentum* specimens examined.

(bolded collectors/numbers are specimens previously cited as paratypes of *S. ossicruentum* by Martine et al. (2016)) **Australia** • **Northern Territory**: Cockatoo Creek, Keep River area, 15°55'17"S, 129°03'31"E, 2 September 1974, ***Gibbs & Fox 618*** (DNA); • Spirit Hills, 15°24'58"S, 129°28'39"E, 17 April 2007, ***Kerrigan 1226*** (DNA); • 11 km east of NE Mt. Frederick, 19°37'S, 129°21'E, 1 March 1981, ***Latz 8597*** (DNA); • 8 km SSW Victoria River Bridge, 15°40'47"S, 131°5'34"E, 16 April 1996, ***Latz 14760*** (DNA); • Cow Creek, Victoria River, Gregory National Park, 15°52'26.8"S, 131°19'58.6"E, 2 May 2001, ***Mangion & Boehme****1060* (DNA); • Nigli Gap Walk, Keep River National Park, 15°45'30.4"S, 129°05'07.4"E, 26 May 2004, ***Martine & Barker 772*** (DNA, CONN); • Gurrundalng Walk, Keep River National Park, 15°52'07.8"S, 129°03'11.1"E, 27 May 2004, ***Martine & Barker 781*** (DNA, CONN); • Bradshaw Military Training Area, 15°04'50"S, 129°33'28"E, 2 April 2007, ***Stuckey & Cowie 64*** (DNA, NSW); • Spirit Hills Conservation Area ca. 1 km W of Nancy’s Gorge, 15°28'19"S, 129°19'58"E, 18 August 1996, *Cowie 7130* (fruit) (PERTH, DNA); • 9 mi. W of East Baines River, 18 June 1967, D.E. Symon 5242 (NSW!, CANB). **Western Australia** • Mirima (Hidden Valley) National Park, below upper lookout on Derdbe-Gerring Banan Lookout Trail, 15°45.827'S, 128°45.105'E, 18 May 2014, ***Martine & Martine 4011*** (DNA, PERTH, BUPL, CONN); • Mornington Station, 17°33'02"S, 132°01'15"E, 11 April 2004, ***Risler & Legge****2673* (DNA); • North end of Ragged Range, 16°31'32"S, 128°23'21"E, 17 July 2001, *Edinger 2601* (DNA, PERTH); • 1 mile N of Revolver Creek, Carr Boyd Ranges, 16°14'S, 128°34'E, 13 March 1978, *Hartley 14561* (DNA); • Mirima National Park, 15°47'14.1"S, 128°45'37.0"E, 28 May 2004, *Martine & Barker* 787 (DNA, CONN); • Carr Boyd Ranges, 16°05.207'S, 128°45.406'E, 3 May 2014, *Martine & Martine 4057* (DNA, BUPL); • Near meatworks on Packsaddle Plain, near Kununurra, 15°57'08"S, 128°28'47"E, 1 March 1993, Mitchell 2822 (flowering) (PERTH); • Kimberleys, S. side of Cockburn Range ca. 6.5 km W. of King River, 10 July 1974, *Carr 33744 & Beauglehole* (flowering) (PERTH); • Hidden Valley near Kununurra, 3 August 1974, *Kenneally 1900* (flowering) (PERTH); • 15 km E of Fitzroy River, Dimond Gorge Rd, 26 June 1975, *Beaugleholei 53908* (flowering) (PERTH); • Northern Carr Boyd Range, 15°56'27.5"S, 128°39'13.2"E, 24 June 2013, *Handasyde 7558* (fruit) (PERTH); • Dillon’s Springs, East Kimberley, October 1906, *Fitzgerald s.n.* (NSW [NSW67613]); Dillon’s Springs, East Kimberley, October 1906, *Fitzgerald s.n.* (NSW [NSW570497]); • Dillon’s Springs, East Kimberley, October 1906, *Fitzgerald s.n.* (AD [AD98581501]); Dillon’s Springs, East Kimberley, October 1906, *Fitzgerald s.n.* (PERTH [PERTH01614207]).

#### Diagnostic couplets.

A comprehensive “Kimberley dioecious clade” key, including newly-recognized species, is forthcoming (Barrett and Barrett in prep). The most complete key to date can be found in Barrett (2013), which lumps the numerous variations of *S. dioicum* sensu lato as a single taxon. The following couplets may be inserted where *S. dioicum* occurs at couplet 60 in the Barrett (2013) key and supplants the single replacement couplet 60a [previously published in Martine et al. (2016) and modified by Williams et al. (2022) to include *S. scalarium*]. Note: The concept of *S. dioicum* in this key remains broad and still includes numerous recognizable variants.

[addition to Barrett 2013; couplet 60]

**Table:** 

60	Veins of abaxial leaf surface with small, round, green-to-black extrafloral nectaries visible to the naked eye	***Solanum nectarifolium* Martine & Brennan**
–	Leaves lacking green-to-black extrafloral nectaries visible to the naked eye	**60a**
60a	Plants with silvery leaf indumentum, overall aspect silvery-blue; typically up to 1–1.5 m tall; few-branched and conspicuously “Y”-shaped in form; stems and calyces heavily armed; stigma shallowly bifid, the lobes 0.5–1 mm long	***Solanum ossicruentum* Martine & J.Cantley**
–	Plants with silvery/rusty/yellow leaf indumentum, overall aspect silvery-green, yellow-green, reddish-green; typically up to 0.5–1 m tall; stigma deeply bifid, the lobes 2–5 mm long	**60c**
60c	Plants many-branched and typically upright in form; stems moderately prickly; leaf indumentum silvery or rusty, overall aspect silvery-green, yellowish green, or reddish green; stigma lobes 2–5 mm long; mature fruits green and fleshy; male floral rachis typically unarmed or lightly armed	***Solanum dioicum* W.Fitzg.**
–	Plants many-branched and spreading decumbent in form; stem densely prickly; leaf indumentum yellow, overall aspect yellow-green; stigma lobes 1.5–2 mm; mature fruits light green to yellow-orange and fleshy, becoming tan and bony hard; male floral rachis armed with straight alternating prickles	***Solanum scalarium* Martine & T.M.Williams**

#### Recircumscription and lectotypification of *Solanum dioicum*.

In 1906, W.V. Fitzgerald collected plants from multiple different solanums at Dillon’s Springs in the eastern Kimberley region of WA. These included two morphologically distinct entities: one he identified as *S. cunninghamii* Benth. (a species described the previous year from the far western Kimberley), and another he recognized as undescribed that he later published as *Solanum dioicum* W.Fitzg. (Fitzgerald 1918) in “The Botany of the Kimberleys, North-western Australia” based on his own field notes and collected material sent to J.H. Maiden in Sydney.

One of the key causes of confusion about the identity of *S. dioicum* is an unintentional error in Fitzgerald’s publication (1918). On page 102, the name *Solanum cunninghamii* appears on both the intended “cunninghamii” record (species #560, saying only that it was a plant about 3 feet tall) and also the *Solanum dioicum* record (species #561, with a more detailed description). Maiden, to his credit, still listed *Solanum dioicum* as a new species found by Fitzgerald at the Dillon’s Springs site in the early pages of the publication. However, the orthographic error led to more than a century of confusion about what specimens should be considered as types for *S. dioicum*.

In 1965, Nerida Ford (NSW), in preparing to send type material to David Symon, added a note to the Dillon’s Springs sheets (interpreted incorrectly as a single gathering and assumed to be of a single species) sent to the Herbarium at the Waite Agricultural Research Institute (ADW) explaining that the five sheets of NSW material (three Fitzgerald had labelled *S. dioicum* and two as *S. cunninghamii*, all without collection numbers) had been combined under one collection number as the type series for the single species, *S. dioicum*. Perhaps influenced by this interpretation of specimens, Symon (1981) also adopted a broad and variable concept of *S. dioicum*, based in part on the variation seen across the five specimens erroneously labelled as holotype and associated isotypes. Symon also recognized that the use and publication of the name “*S. cunninghamii*” in the text in place of *S. dioicum* by Fitzgerald represented an error and also noted that the “cunninghamii” material was not *S. cunninghamii* but just another version of *S. dioicum*—thus establishing a very variable definition of *S. dioicum* based on the putative set of type specimens (in agreement with Symon’s groups as described above). Symon (1981) did, however, list one of the three non-“cunninghamii” sheets (a staminate specimen and the only one with a label that includes “sp. nov.”) as the “holotype” for *S. dioicum*. This constitutes effective lectotypification, as the original protologue did not mention a holotype, and Symon (1981) cited a single specimen in a single herbarium.

Prompted by R. Barrett’s (NSW) recognition that the five herbarium sheets likely represented distinct plants and indeed distinct species, we examined all five putative type specimens and redetermined the two “cunninghamii” sheets as *Solanum ossicruentum*. Those sheets (and their probable duplicates at AD [AD98581501] and PERTH [PERTH01614207]), which represent the only known records for *S. ossicruentum* from Dillon’s Springs, are not to be considered isotypes of *S. dioicum* and are listed here as specimens examined under *S. ossicruentum*. This correction resolves a key source of ambiguity in the application of the names *S. dioicum* and *S. ossicruentum*. More work is required, however, to now better define the taxonomic identity of *S. dioicum* sensu stricto based on the lectotype as cited below.

### ﻿ Solanum dioicum

Taxon classificationPlantaeSolanalesSolanaceae

W.V. Fitzg., Journal of the Royal Society of Western Australia (1916–1917, issued 1918) 203

6BE723DF-B84C-5CDD-BB7F-04CC73DAF059

#### Type.

Australia • Western Australia: Dillon’s Springs, 15.9247°S, 128.35142°E (estimated), October 1906 (stam fl), *W.V. Fitzgerald s.n.* (lectotype, designated by Symon 1981, pg. 288 [as”holotype”]: NSW [NSW570345, specimen with label information “*Solanum dioicum* W.V.F. sp. nov. Dillon’s Springs, E Kimberley.” Probable isolectotypes BM [BM000846754], E [E00190702], NSW [NSW570336], NSW [NSW570337], PERTH [PERTH01614215].

## ﻿Conclusion

*Solanum nectarifolium* is one of a number of functionally dioecious species from the “Kimberley dioecious clade” that have been described in the last two decades (see Martine et al. 2011; Barrett 2013; Martine et al. 2013; Martine et al. 2016; Williams et al. 2022). Despite this taxonomic effort, *S. dioicum* as currently circumscribed still consists of numerous forms that are candidates for recognition at the species level (Barrett 2013). Notably, none of these variants and none of the already described species in the clade bear visible extrafloral nectaries on the leaves. This character appears to be unique among the 1,200+ species of *Solanum* that are currently described.

## Supplementary Material

XML Treatment for Solanum nectarifolium

XML Treatment for Solanum dioicum

## ﻿Additional information

### Use of AI

No use of AI was reported.

### Author ORCIDs

Christopher T. Martine https://orcid.org/0000-0001-5143-1773

Jason T. Cantley https://orcid.org/0000-0001-7748-0397

Aiden T. Webb https://orcid.org/0009-0007-0344-5805

### Data availability

All of the data that support the findings of this study are available in the main text or Supplementary Information.

## Supplementary materials

Supplementary material 1 Accessions of *Solanum dioicum* and *S. ossicruentum* held in Australian herbaria attributable to the new species *Solanum nectarifolium* Martine & Brennan Data type docx Explanation note Rows with more than one current identification are for collections filed under different names depending on the herbarium where they are held, as per the Australasian Virtual Herbarium (15 Oct 2025). https://binary.pensoft.net/file/1501905This dataset is made available under the Open Database License (http://opendatacommons.org/licenses/odbl/1.0/). The Open Database License (ODbL) is a license agreement intended to allow users to freely share, modify, and use this Dataset while maintaining this same freedom for others, provided that the original source and author(s) are credited.Christopher T. Martine, Kym Brennan, Jason T. Cantley, Aiden T. Webb, Geoffrey Newton
