# Supplementary material for: ﻿A new dioecious bush tomato, Solanum nectarifolium (Solanaceae), from the northern Tanami Desert, Northern Territory, Australia, with reassessment of S. ossicruentum and a change in the circumscription of S. dioicum
Source: PhytoKeys. 2025 Dec 29;268:183–99. doi: 10.3897/phytokeys.268.169893 (PMC12770996; doi:10.3897/phytokeys.268.169893)
Supplement: Supplementary material 1 — Accessions of Solanum dioicum and S. ossicruentum held in Australian herbaria attributable to the new species Solanum nectarifolium Martine & Brennan [file phytokeys-268-183_article-169893__-s001.docx]

| **Current ID in herbarium** | **Collector** | **Collection year** | **Collector number** | **Herbarium(a)** | **Locality** |
| --- | --- | --- | --- | --- | --- |
| *S. dioicum*/*S.ossicruentum* | J.R. Maconochie | 1971 | 1122 | CANB, DNA, NT, PERTH | 37 miles SW of Hookers Creek |
| *S. dioicum*/*S.ossicruentum* | D.E. Symon | 1971 | 6937/6938 | AD, CANB, DNA, NT, PERTH | 37 miles SW of Hookers Creek |
| *S. dioicum*/*S.ossicruentum* | P.K. Latz | 2003 | 19451 | AD, NT | Winnecke Hills |
| *S. dioicum*/*S.ossicruentum* | C.H. Gittins | 1971 | 2401 | BRI, CANB, NSW | Gardner Range, 80-96 km NW of Tanami |
| *S. dioicum*/*S.ossicruentum* | C.H. Gittins | 1971 | 2396 | BRI, NSW | Gardner Range, 81-97 km NW of Tanami |
| *S. dioicum*/*S. ossicruentum* | P.K. Latz | 1973 | 4019 | AD, DNA, PERTH | Sturt Creek Station, Denison Range |
| *S. ossicruentum* | K. Brennan | 2021 | 12086 | DNA | 48 km SW of Lajamanu |
| *S. ossicruentum* | C.P. Manion and D.L. Lewis | 2004 | 1607 | DNA | Winnecke Hills |
| *S. ossicruentum* | T.M. Orr | 1988 | 57 | DNA | 63 km S of Lajamanu |
| *S. ossicruentum* | P.K. Latz | 1981 | 8597 | DNA | 11 km ENE of Mt. Fredrick |
| *S. ossicruentum* | D.E. Albrecht | 1996 | 7756 | DNA, NT | Jellebra Rockhole |

**Supplementary Table 1.** Accessions of *Solanum* *dioicum* and *S*. *ossicruentum* held in Australian herbaria attributable to the new
species *Solanum nectarifolium* Martine & Brennan. Rows with more than one current identification are for collections filed under different names depending on the herbarium where they are held, as per the Australasian Virtual Herbarium (15 Oct 2025).
